# Trabeculopuncture as a predictive test of distal outflow resistance in canal-based surgery

**DOI:** 10.1038/s41598-022-13990-9

**Published:** 2022-06-22

**Authors:** R. Verma-Fuehring, M. Dakroub, H. Han, J. Hillenkamp, N. A. Loewen

**Affiliations:** 1 Department of Ophthalmology, University of Würzburg, Würzburg, Germany; 2 Artemis Eye Centers of Frankfurt, Hanauer Landstraße 147-149, 60314 Frankfurt, Germany

**Keywords:** Eye diseases, Glaucoma

## Abstract

We investigated whether trabeculopuncture (TP) could detect distal outflow resistance to predict the outcome of canal-based glaucoma surgery such as ab interno trabeculectomy (AIT). These procedures have a high utilization in open angle glaucoma, but fail in eyes with an unidentified distal outflow resistance. We assigned 81 porcine eyes to two groups: trial (n = 42) and control (n = 39). At 24 h, four YAG-laser trabeculopunctures were placed nasally, followed by a 180° AIT at the same site at 48 h. The proportion of TP responders between both AIT groups was compared. Histology and outflow canalograms were determined. Both post-TP and post-AIT IOPs were lower than baseline IOP (*p* = 0.015 and *p* < 0.01, respectively). The success rates of TP and AIT were 69% and 85.7%, respectively. Sensitivity and specificity values of TP as predictive test for AIT success were 77.7% and 83.3%, respectively. The positive and negative predictive values were 96.6% and 38.5%, respectively. We conclude that a 10% reduction in IOP after TP can be used as a predictor for the success (> 20% IOP decrease) of 180° AIT in porcine eyes.

## Introduction

Intraocular pressure (IOP) reduction is the only treatment for glaucoma^1^ demonstrated to be effective with high-quality level I evidence^2–4^. Selective laser trabeculoplasty, now a recommended first line of treatment for most open angle glaucomas^5^, and medications may achieve the desired IOP levels in many patients, but about 50% still require surgery eventually^6^. Traditional glaucoma filtering surgeries^7^ lower the IOP by bypassing the trabecular meshwork (TM) and draining the aqueous humor into a newly created epibulbar space^8^, but they are associated with a high rate of complications and require intensive postoperative care^9,10^.

Canal-based minimally invasive glaucoma surgeries produce far fewer complications and allow intervening earlier^11,12^ because they lower the IOP by bypassing or removing the TM to enhance the physiological aqueous humor outflow route^11,13,14^. Leading modalities are trabecular bypass stents (TBS)^15–17^ and ab interno trabeculectomy (AIT), in which the TM is either ablated^18^, incised^19^, or excised^20^. However, approximately 30% of patients experience an insufficient IOP reduction^21^. One would expect the IOP to be close to the level of episcleral venous pressure, approximately 8 mmHg, but this pressure can rarely be achieved^21^ due to increased post-trabecular resistance^22,23^. So far, there is no presurgical test that could assess the post-trabecular resistance predict the outcome of AIT.

A noninvasive procedure that allows aqueous to bypass the proximal outflow resistance at least temporarily is Nd:YAG laser-assisted trabeculopuncture (TP). Not unlike trabecular bypass stents, TP creates a focal opening through the trabecular meshwork (TM) and the inner wall of Schlemm’s canal (SC)^24^. In 1985, Epstein et al. investigated TP as a treatment for glaucoma, but the subsequent IOP reduction was short-lived^24^.

Here, we hypothesized that TP could be used to assess the distal outflow tract function before AIT or TBS are considered. We deployed our porcine anterior chamber ex vivo perfusion model^25,26^ to develop a predictive test and address this unmet need.

## Methods

### Study design

In total, 81 hemisected, perfused porcine eyes were assigned to one of two groups: trial (T) (n = 42) and control (C) (n = 39). Eyes in the trial group underwent trabeculopuncture using a Nd:YAG laser 24 h after incubation, followed by ab interno trabeculectomy a day later. The IOP was measured continuously for 72 h, with baseline values being recorded 24 h (IOP_BL_) after the start of the experiment. Post-trabeculopuncture IOP (IOP_TP_) was measured at 48 h, and post-ab interno trabeculectomy IOP (IOP_AIT_) at 72 h. The estimated outflow facility (C) was calculated by dividing the medium inflow by the IOP value. The fluid inflow simulated aqueous humor formation and was constantly at 6 µl/min.

The eyes in the control group did not undergo any procedures but were incubated and monitored similarly for 72 h. No live vertebrate animals were used to conduct this study.

### Preparation and Incubation

Freshly enucleated porcine eyes were obtained from a local abattoir (Landschlachterei Issing, Retzbach, Bavaria, Germany) and processed within three hours postmortem. Institutional Animal Care and Use Committee review was waived because animals were not being sacrificed for the purpose of doing research. All eyes were stripped of extra-orbital tissue, placed in a 2.5% povidone-iodine solution for 30 s, and rinsed with phosphate-buffered saline (PBS). After bisecting the eyes at the equator, the vitreous body, lens, and uvea were removed in one piece. The anterior segments were mounted in custom-made perfusion dishes, incubated at 37 °C, and perfused with Dulbecco’s Modified Eagle’s Medium (DMEM) fortified with Penicillin/Streptomycin at a rate of 6 µl/min using a perfusion pump (Harvard PHD ULTRA™ CP Syringe Pump, Harvard Apparatus, Holliston, MA, USA). The dishes were connected to pressure transducers (Daltren DPT-200, Utah Medical Products Inc., Midvale, USA) which provided continuous IOP measurements at a rate of one reading every 2 min using LabChart (Version 8.1.16, ADInstruments, Sydney, Australia).

### Nd:YAG-Laser TP and AIT

After perfusion for 24 h, the anterior segments were removed from the dish, and four evenly spaced trabeculopunctures were placed along the nasal 180° of trabecular meshwork using a Q-switched Nd:YAG laser (VISULAS YAGIII, Zeiss, Oberkochen, Germany). Fifteen shots with an energy of 7–10 mJ were applied for each puncture. A 180-degree ab interno trabeculectomy was performed 24 h later along the same nasal 180° of the trabecular meshwork^26–28^.

### Canalograms

After an anterior chamber exchange, eyes were placed under a stereomicroscope (Olympus SZX, Olympus K.K., Tokyo, Japan) for a baseline canalogram using a gravity-driven infusion of fluorescent microspheres (1:25 dilution, FluoSpheres 0.5 µm, Thermo Fisher Scientific Inc., Waltham, MA, USA) for 10 min. Canalograms were recorded and analyzed (cellSens Dimension, version 2.3, Olympus K.K., Tokyo, Japan) both after TP and AIT.

### Histology

We obtained sagittal sections before TP as well as after TP and AIT and fixed them with 4% paraformaldehyde in PBS for 24 h. After rinsing them three times in PBS, they were embedded in paraffin, sectioned at 6-micron thickness, and stained with hematoxylin and eosin.

### Statistical analysis

Our sample size calculation indicated a minimum requirement of 35 eyes per group to achieve a testing power of 0.9. We analyzed data with SPSS Statistics (Version 26, IBM, New York, USA). Means and standard deviations were reported for all parameters. We tested for normal distribution with the Kolmogorov–Smirnov test and used a paired t-test or a Wilcoxon Signed Rank test to compare dependent means; an unpaired t-test or Mann–Whitney U test was used for independent means. One-way repeated measures multivariate analysis of variances (MANOVA) was used to compare more than two means. The Pearson Coefficient was reported for the correlation between continuous variables. TP and AIT success was defined as a decrease of 5% and 10% from baseline IOP, respectively. We used Fisher’s exact test to compare the number of eyes that responded to TP and AIT to the number of eyes that responded to TP but failed AIT. Sensitivity, specificity, positive predictive value (PPV), and negative predictive value (NPV) were calculated. After TP and AIT success, a receiver operating characteristic curve (ROC curve) was plotted for IOP reduction. A* p* value of 0.05 or less was considered statistically significant for all tests.

## Results

**Figure 1:**
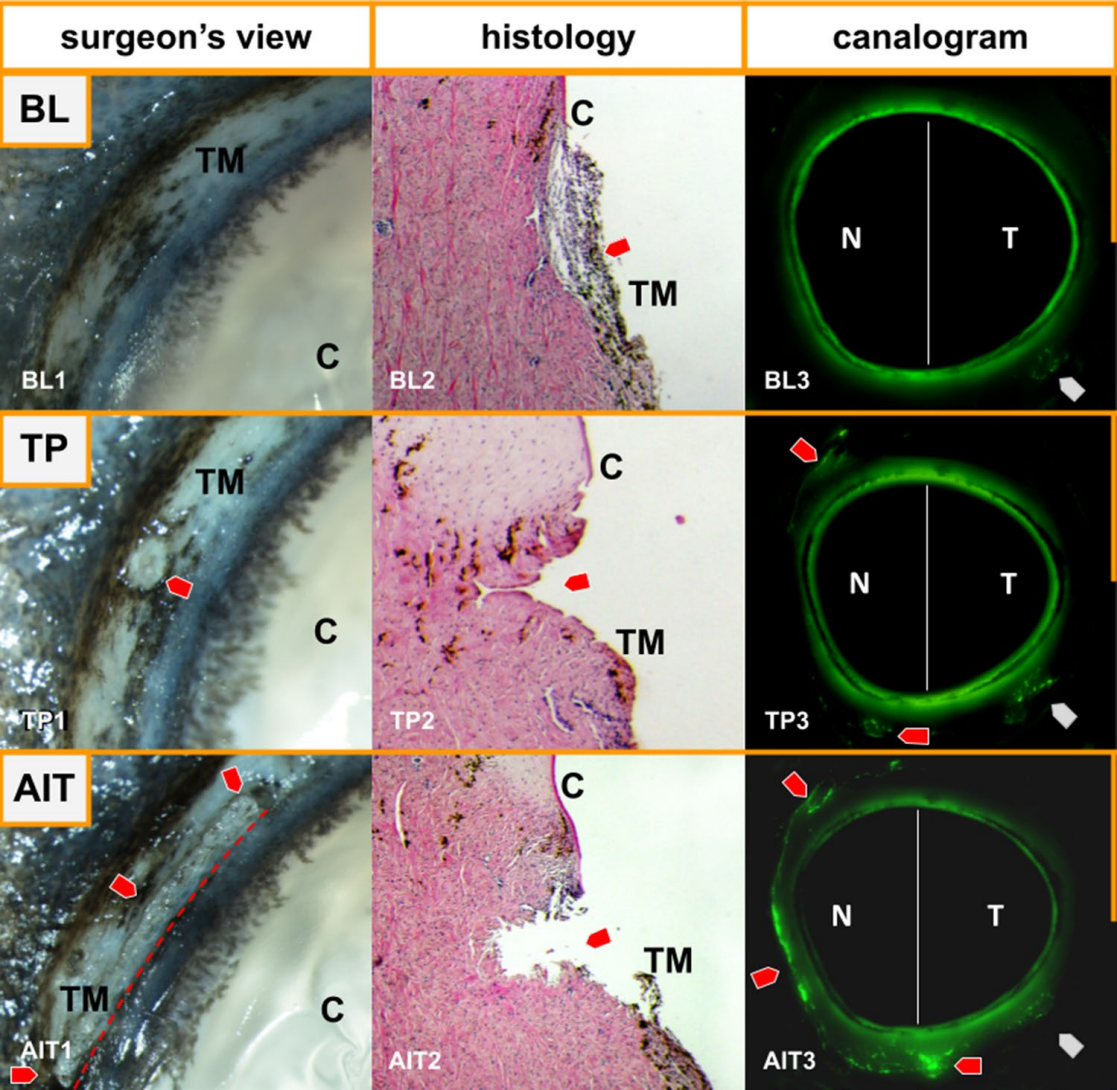
Visualization of the effects of trabeculopuncture (TP) and ab interno trabeculectomy (AIT). Microscopic images, histology sections, and canalograms at baseline (BL), after trabeculopuncture (TP), and after ab interno trabeculectomy (AIT). BL1: Microscopic image of the porcine trabecular meshwork at baseline. BL2: Histological analysis of a section of the intact porcine TM (red arrow). BL3: Canalogram image of an anterior segment perfused with fluorescent spheres. No sphere collection can be seen in the nasal episcleral veins at baseline. Single beads were detected in the temporal region (gray arrow). TP1: Ex vivo image of a nasal YAG-TP. TP2: Histological analysis of a section after TP on the TM (red arrow). TP3: Canalogram image of an anterior segment perfused with fluorescent spheres after TP. Fluorescent spheres can now be visualized in the nasal episcleral veins (red arrows). Temporal fluorescence remained unchanged from baseline (gray arrow). AIT1: Image post trabeculectomy. The red arrows outline the excision in the TM. AIT2: Histological section after AIT. The trabecular meshwork is excised (red arrow). AIT3: Canalogram image of an anterior segment perfused with fluorescent spheres. An increased accumulation of spheres along the nasal circumference can be seen (red arrows). TM = trabecular meshwork; C = cornea; N = nasal; T = temporal.

Pilot experiments with whole eyes showed that a transcorneal TP using a Ritch trabeculoplasty lens could not be accomplished reliably. Therefore, anterior segments were inverted to laser the TM directly. The TM could be readily identified (Fig. 1, BL1). The procedure resulted in small, well-circumscribed pits of approximately 500 µm in length and 250 µm in depth (Fig. 1, TP1). We detected no obvious damage to adjacent tissue upon inspection with a microscope. AIT removed the TM extensively, leading to a narrow continuous groove along the nasal quadrants (Fig. 1, AIT1). After both procedures, the findings on histological sections (Fig. 1, middle row) corresponded well to our observations through an operating microscope. These were further supported by canalograms (Fig. 1, BL3), which illustrated an improved localized outflow after TP (Fig. 1, TP3) and further increased sectoral outflow in the nasal quadrants and adjacent drainage segments after AIT (Fig. 1, AIT3).

**Figure 2:**
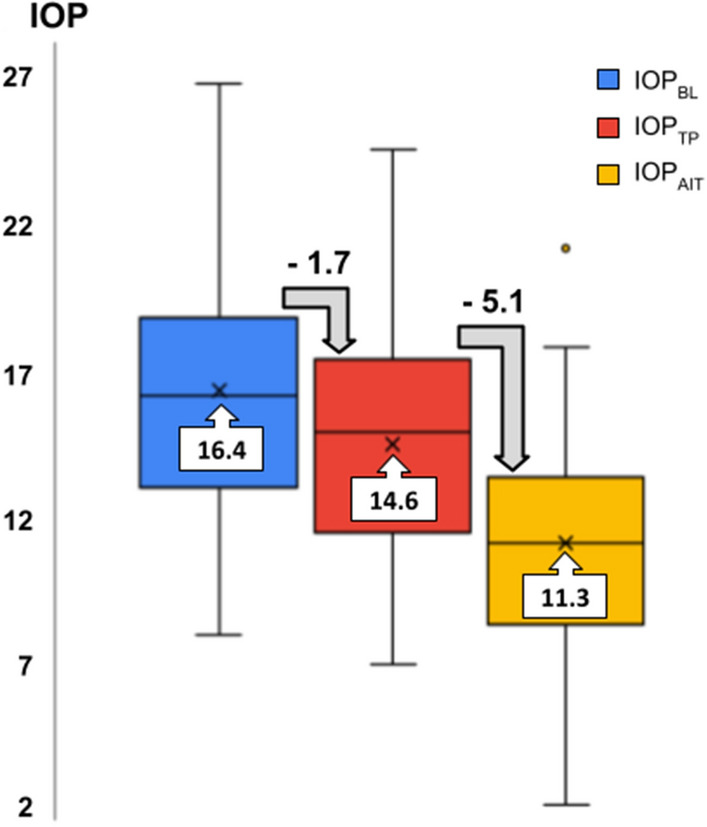
Intraocular pressure (IOP) levels at baseline and post intervention. Boxplots for mean IOP levels at Baseline (IOP_BL_), post trabeculopuncture (IOP_TP_), and post ab interno trabeculectomy (IOP_AIT_). The figures above the grey arrows correspond to the mean IOP reduction after trabeculopuncture and ab interno trabeculectomy, respectively.

Eighty-one eyes were analyzed: 42 trial eyes (T) and 39 controls (C). Table 1 depicts the IOP parameters of both groups. Baseline IOP (IOP_BL_) was 16.4 ± 4.5 mmHg in T eyes and 15.2 ± 3.9 mmHg in C eyes. There was no difference between both variables (*p* = 0.37). In the experimental group, mean IOP_TP_ and IOP_AIT_ values were 14.6 ± 4.3 mm Hg and 11.3 ± 3.8 mmHg, respectively. Both values were lower than IOP_BL_ (*p* = 0.02 and *p* < 0.001, for IOP_TP_ and IOP_AIT_, respectively). We found the three IOP measurements (IOP_BL_, IOP_TP,_ and IOP_AIT_) to be different from each other (*p* < 0.001). Figure 2 illustrates the mean IOP levels of all three groups and their respective average post-procedure IOP drops. The average IOP reduction from IOP_BL_ after TP and AIT was 8.7 ± 22.4% and 28.8 ± 22.8%, respectively. There was a weak positive correlation between the amount of IOP reduction from IOP_BL_ after TP and AIT (r = 0.37, *p* = 0.015). Control eyes had an IOP that was 2.0 ± 1.3 mmHg higher at the end of the perfusion studies (*p* > 0.05 for both 24 h and 48 h).

**Table 1:** IOP parameters of trial and control group.

Group	Parameter	IOP-Value (mmHg)	Diff from IOP_BL_ (mmHg)	*p* value for IOP_BL_ reduction	*p* value for Trial versus Control
Trial (n = 42)	IOP_BL_	16.4 ± 4.5	–	–	0.37
	IOP_TP_	14.6 ± 4.3	− 1.7 ± 3.8	0.015*	0.16
	IOP_AIT_	11.3 ± 3.8	− 5.1 ± 4.4	< 0.001*	< 0.001*
Control (n = 39)	IOP_BL_	15.2 ± 3.9	–	–	0.37
	IOP_48_	15.9 ± 4.0	0.8 ± 1.6	0.01*	0.16
	IOP_72_	17.3 ± 4.6	2.0 ± 1.3	< 0.001*	< 0.001*

IOP_BL_ = baseline IOP after 24 h; IOP_TP_ = IOP 24 h after trabeculopuncture; IOP_AIT_ = IOP 24 h after ab interno trabeculectomy; IOP_48_ = IOP after 48 h of incubation; IOP_72_ = IOP after 72 h of incubation.

**Figure 3:**
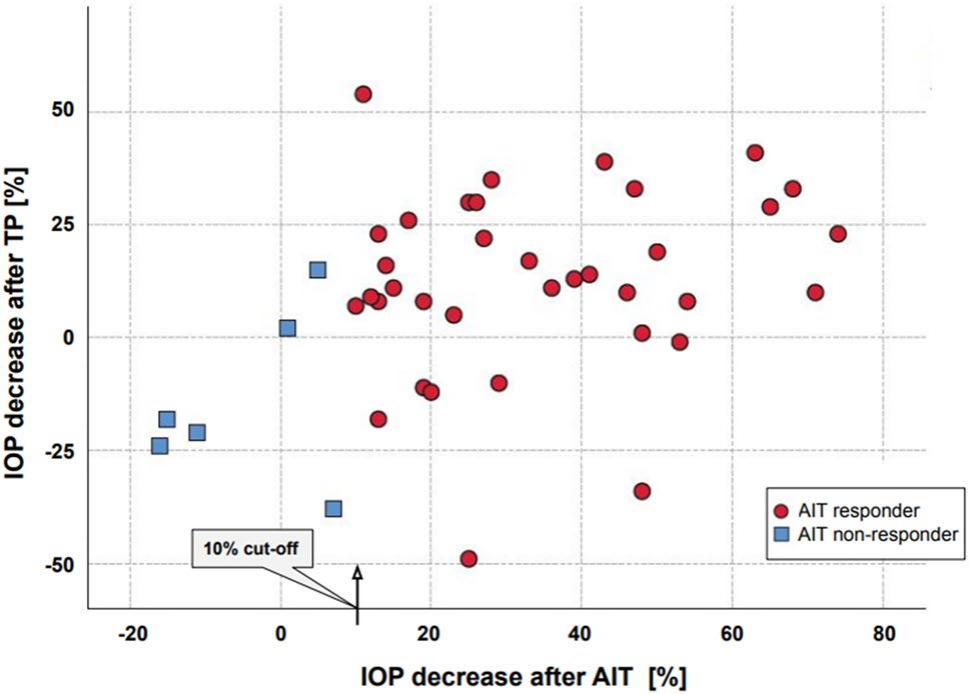
Scatter Plot of IOP variation after trabeculopuncture and ab-interno trabeculectomy. IOP alteration after trabeculopuncture (TP) and ab-interno trabeculectomy (AIT) are plotted. Negative percentages indicate an increase in IOP. AIT responders (red circles) and non-responders (blue boxes) can be distinguished. The cut-off for AIT success was set to 10%.

**Figure 4:**
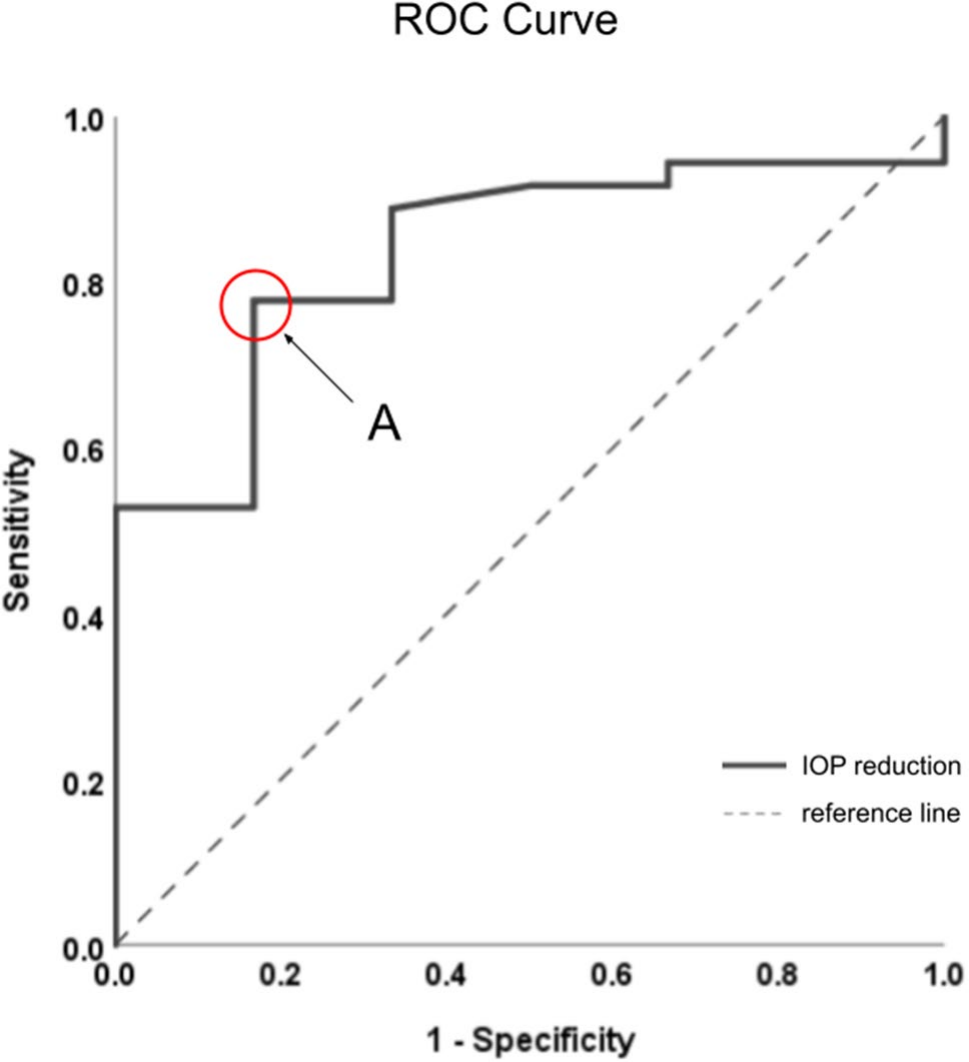
Receiver operating characteristics (ROC) curve for post-trabeculopuncture IOP reduction in detecting ab interno trabeculectomy responders. Point A corresponds to a 5% IOP drop (resembling a 10% IOP reduction) after trabeculopuncture representing a sensitivity of 76.4% and specificity of 83.3%.

Figure 3 plots all 42 eyes of the trial group according to IOP change after TP and AIT, respectively. After TP and AIT success, it visualizes that four out of six non-responders to AIT show an IOP increase after TP. Table 2 depicts the distribution of TP responders in both AIT responders and AIT non-responders for all tests. The total number of TP responders was 29 (69%); for AIT, this value was 36 (85.7%). The proportion of TP responders among AIT responders was greater than that among AIT non-responders (66.7% vs. 19%, respectively, *p* = 0.007). TP's positive and negative predictive values as a test for predicting AIT success were 96.6% and 38.5%, respectively. Sensitivity and specificity values were 77.7% and 83.3%, respectively. This combination of values was plotted as a ROC curve of a 5% post-TP IOP drop, predicting AIT success (Fig. 4).

**Table 2:** Distribution of AIT and TP responders and non-responders.

Outcome	AIT responders (n, %)	AIT non-responders (n, %)	Total (n, %)
TP responders (n, %)	28 (66.7%)	1 (2.4%)	29 (69.0%)
TP non-responders (n, %)	8 (19.0%)	5 (11.9%)	13 (31.0%)
Total (n)	36 (85.7%)	6 (14.3%)	42 (100%)

AIT = ab interno trabeculectomy. TP = YAG-assisted Trabeculopuncture.

A subanalysis (Supplementary Information, Table 1) showed a mean IOP_BL_ of 17.1 ± 4.4 mmHg and 11.9 ± 2.7 mmHg for AIT responders and AIT non-responders. These values differed significantly (*p* = 0.008). There was no difference in IOP_TP_ and IOP_AIT_ in both subgroups (*p* = 0.48 and *p* = 0.45, Supplementary Table 1). 1Out of all 13 TP non-responders, 76.9% (n = 10) showed an IOP increase after TP of at least 10%.

## Discussion

In this paper, we used a porcine anterior segment model to assess the utility of TP in predicting the success of AIT. The lack of good predictive tests combined with the relatively high rate (30%) of canal-based surgeries and implants led us to explore simple options to avoid unnecessary surgeries. Porcine eyes have been used as a model to study glaucoma extensively and were used here for that reason^29,30^.

We expected TP to cause an IOP drop in our porcine anterior segment perfusion model, similar to that reported in human eyes. TP is not unlike trabecular bypass stents, which also cause a focal opening in the TM and increase outflow in our model^17^. The total energy used was higher than what is normally required in humans due to an at least three times thicker TM compared to human eyes^30^. After AIT, a further decrease in IOP was seen because of the comprehensive excision of the nasal TM. Compared to a study by Dang et al., which tested outflow enhancements of three different AIT devices on porcine eyes, we found similar baseline IOP values (16.35 ± 4.52 mmHg vs. 15.93 ± 2.08 mmHg)^26^. Still, we observed a post-AIT IOP decreased by only 31%, in contrast to the 48% reported in that study^26^.

The control group in our cohort experienced a small IOP increase of approximately 13% over 72 h. This is in line with Dang et al., who observed a 10% IOP increase in control eyes during 72 h of incubation^31^. To adjust for this, we chose 5% and 10% to be satisfactory post-TP and post-AIT IOP reductions, respectively. These values correspond to a 10% and 20% IOP reduction after these procedures. Clinically, a 20% post-AIT IOP reduction is often regarded as sufficient for mild to moderate glaucoma^32^. Being able to predict this outcome will help avoid unnecessary procedures and decrease the burden on the healthcare system.

Interestingly, our subanalysis (Supplementary Information, Table 1) revealed that AIT responders had a higher baseline IOP compared to non-responders. This is perhaps to be expected, as AIT generally caused a greater decrease in eyes with higher baseline IOPs in clinical studies^33^. After TP, AIT non-responders also had a higher mean IOP resembling a decreased outflow facility, respectively, which did not reach significance with the number examined here. It is possible that the collapse of laser-induced trabeculopunctures temporarily decreases the outflow facility as described before^34,35^, which could similarly affect our TP non-responders if their TM was compromised in the area of the TP and AIT. Additionally, there was no difference between baseline and post-AIT IOP levels in AIT non-responders, which is indicative of a post-trabecular meshwork resistance in these eyes. These were not glaucomatous eyes, however. Ocular hypertension can be induced experimentally in pig eyes^36^ but does not occur in pigs naturally. We suspect that inadvertent compression of key elements of the distal outflow tract in the nasal quadrants by the compression ring of the perfusion mount is responsible for this. This would not necessarily lead to an increased IOP because at least 3/4th of the outflow tract has to be compromised^37^, but it can explain the failure to respond to TP and AIT. However, one has to be careful interpreting the results of this subanalysis, as there were only six AIT non-responders in our study with a significantly lower baseline IOP than AIT responders.

A simple and noninvasive predictive test for canal-based surgeries that ablate, excise, disrupt or bypass the TM is urgently needed because of the rapidly increasing demand for these procedures. The implementation of a Nd:YAG laser-TP for such a test is straightforward as this device is ubiquitously available in ophthalmology practices and clinics, and most ophthalmologists are familiar with its use^38^. Although the effect of TP is too short-lived to be useful for glaucoma treatment^39,40^, it is precisely this benign nature that may afford a low-risk test of distal outflow resistance.

The amount of IOP reduction after TP and AIT had a relatively weak direct correlation. This is not surprising and matches the clinical reality that AIT will lower IOP, not by a certain percentage that can be generalized regardless of baseline IOP. Instead, AIT lowers IOP to a pressure level defined by residual, post-trabecular outflow resistance and independent of baseline IOP. For instance, both an eye with a pressure of 35 mmHg and another eye with a pressure of 20 mmHg can have a similar postoperative IOP because the primary resistance at the level of the trabecular meshwork is removed. Therefore, the weak correlation in our study does not undermine the utility othanpredictive test with a high PPV for the success of AIT. Moreover, our sensitivity and specificity values of 77% and 83% are sufficient for a clinical test. However, our data did not show a high negative predictive value (NPV, 38.5%) in porcine eyes because these are non-glaucomatous eyes. The NPV in human eyes should be higher, matching the AIT failure rate caused by a presumed higher rate of post-trabecular resistance than in pigs.

One limitation of our study is the ex-vivo setting. Hence, wound healing of the TM and its effect on IOP cannot be observed. Another limitation is the anatomical difference between porcine eyes and human eyes. In porcine eyes, the outflow tract consists of an angular aqueous plexus, whereas humans have a Schlemm’s canal, often with a single lumen^41,42^. We used four evenly spaced TPs over the nasal angle to cover the extent of an AIT and to account for the decreased circumferential flow compared to a human Schlemm’s canal. Clinically, blood reflux from SC can normally be seen after a TP, a useful indicator of completion absent in an ex vivo model. Instead, we had to use the IOP decrease and an increased outflow of fluorescent beads as an indicator. We did not perform canalograms with bead tracers on eyes whose IOP was obtained because they can progressively obstruct distal outflow channels and alter IOP measurements. Therefore, we could not compare the outflow patterns of responders and non-responders. Our study was not designed, and hence not powered, to discover a cause of AIT failure. It could be possible that a post-trabecular outflow resistance in the form of the compression mount of the culture dish causes the inability to achieve an improved facility by TP and AIT. For instance, a decentered mount may compress a part of the distal outflow system in the area treated with TP and AIT but not affect other parts of the circumference. As a result, the outflow at baseline would not be impaired because more than 75% of the TM has to be blocked in this model for IOP to rise^37^. On the other hand, debris created by TP or AIT may slightly reduce outflow away from the treated site precisely as we have observed in those eyes but not in others. Investigating the mechanism is relevant to past and future studies using perfused anterior segments and requires a study specifically designed to test this hypothesis.

In conclusion, a 10% IOP reduction after trabeculopuncture can be used to predict a successful ab interno trabeculectomy in porcine eyes.

## Supplementary Information

Supplementary Information.

## Data Availability

Data is readily available from the corresponding author on reasonable request.
