# Supplementary material for: Trabeculopuncture as a predictive test of distal outflow resistance in canal-based surgery
Source: Sci Rep. 2022 Jun 22;12:10584. doi: 10.1038/s41598-022-13990-9 (PMC9218089; doi:10.1038/s41598-022-13990-9)
Supplement: Supplementary file 1 — Supplementary Information. [file 41598_2022_13990_MOESM1_ESM.pdf]

Trabeculopuncture as a predictive test of distal outflow resistance in canal-based surgery

Supplementary Information

Verma-Fuehring R[1], Dakroub M[1], Han H[1], Hillenkamp J[1], Loewen NA[1,2*]

1. Department of Ophthalmology, University of Würzburg, Würzburg, Germany
2. Artemis Eye Centers, Frankfurt, Germany

Correspondence to:

Nils Loewen, MD, PhD
Department of Ophthalmology,
University Hospital Würzburg
Josef-Schneider-Straße 11
97080 Würzburg
Germany
loewen.nils@gmail.com

Supplementary Information

Supplementary Table 1

Supplementary Table 1 Subanalysis of the trial group in AIT and TP success, respectively. Positive values represent an increase in IOP (in mmHg) or estimated outflow facility (C, in µl/min*mmHg). C is defined as inflow/IOP. The inflow was constantly at 6µl/min. Delta represents the difference between post-interventional and baseline values.
TP = Trabeculopuncture; AIT = ab interno trabeculectomy; IOP_{BL} = baseline IOP; IOP_{TP} = IOP 24 hours after trabeculopuncture; IOP_{AIT} = IOP 24 hours after ab interno trabeculectomy; C_{BL} = baseline outflow facility; C_{TP} = outflow facility 24 hours after trabeculopuncture; C_{AIT} = outflow facility 24 hours after ab interno trabeculectomy

Group	Variable	Delta	Value	p-value for Difference from Baseline
**AIT responders (n = 36)**	IOP_{BL}<br>IOP_{TP}<br>IOP_{AIT}	 -2.3<br>-6.0	17.1 ±4.4<br>14.8 ±4.5<br>11.1 ±3.9	 < 0.01*<br>< 0.01*
	C_{BL}<br>C_{TP}<br>C_{AIT}	 0.08<br>0.27	0.37 ±0.10<br>0.45 ±0.16<br>0.64 ±0.36	 < 0.01*<br>< 0.01*
**AIT non-responders (n = 6)**	IOP_{BL}<br>IOP_{TP}<br>IOP_{AIT}	 1.5<br>0.4	11.9 ±2.7<br>13.5 ±3.4<br>12.4 ±2.6	 0.53<br>1.0
	C_{BL}<br>C_{TP}<br>C_{AIT}	 -0.6<br>-0.3	0.53 ±0.13<br>0.47 ±0.10<br>0.50 ±0.10	 0.42<br>0.96
**TP responders (n = 29)**	IOP_{BL}<br>IOP_{TP}<br>IOP_{AIT}	 -3.6<br>-6.1	17.6 ±4.7<br>14.0 ±4.3<br>11.5 ±4.2	 < 0.01*<br>< 0.01*
	C_{BL}<br>C_{TP}<br>C_{AIT}	 0.11<br>0.27	0.37 ±0.11<br>0.48 ±0.17<br>0.64 ±0.41	 < 0.01*<br>< 0.01*
**TP non-responders (n = 13)**	IOP_{BL}<br>IOP_{TP}<br>IOP_{AIT}	 -2.5<br>2.7	13.6 ±2.6<br>16.1 ±4.0<br>10.9 ±2.5	 0.01*<br>0.04*
	C_{BL}<br>C_{TP}<br>C_{AIT}	 -0.06<br>0.12	0.46 ±0.11<br>0.40 ±0.10<br>0.58 ±0.13	 < 0.01*<br>0.048*
